# Temporal trend of hospitalization for chronic venous disease in Brazil from 2014 to 2023

**DOI:** 10.1590/1677-5449.202401362

**Published:** 2025-10-27

**Authors:** Lucas Sgrott Simão Flausino, Douglas Fernando Kunkel, João Guilherme Cardoso, Lucas Napoli Cordeiro, Kailan Goulart da Silveira, Gabriele Sousa Silval, Maysa Melo Henkes, Fabiana Oenning da Gama

**Affiliations:** 1 Universidade do Sul de Santa Catarina – UNISUL, Palhoça, SC, Brasil.

**Keywords:** vascular diseases, varicose veins, thrombophlebitis, thrombosis, hospitalization, epidemiology

## Abstract

**Background:**

Chronic Venous Disease (CVD) of the lower extremities (LEs) is a condition with significant prevalence and hospital admission rates.

**Objectives:**

To analyze temporal trends in hospitalization due to CVD from 2014 to 2023 in Brazil.

**Methods:**

An ecological time series study of CVD-related hospitalization using data from the Brazilian Unified Health System. Data were analyzed using simple linear regression with annual variation (β) and percentage changes, with significance set at p < 0.05.

**Results:**

A total of 1,091,733 CVD-related hospital admissions were recorded for the period assessed. The overall rate showed a stable trend (β = -3.233; p = 0.060), with an average of 73.98 hospital admissions per 100,000 inhabitants. Statistically significant reductions were observed in the Northeast (β = -1.275; p = 0.042), Southeast (β = -4.262; p = 0.044), and South (β = -7.282; p = 0.012) regions of Brazil. The North and Midwest regions had stable rates. Both sexes showed stable trends, although hospital admission rates were higher among women. Significant reductions were found among men aged 20 and older and among women aged 20 - 49 and 70 and older. Stable rates were observed in women aged 50-69.

**Conclusions:**

Overall hospital admission rates for CVD in Brazil remained stable between 2014 and 2023. However, regional and age-specific differences highlight the need for targeted public health strategies, particularly for women aged 50-69 and residents of the North and Midwest regions.

## INTRODUCTION

Lower extremity (LE) chronic venous disease (CVD) stands out as one of the chronic diseases with greatest prevalence and incidence, causing elevated morbidity in the population.^1,2^

CVD is defined as a dysfunction of the venous system caused by valve incompetence combined or not with obstruction of venous flow. This venous abnormality may be congenital or acquired and can involve both the superficial and deep vein systems.^3,4,5^

According to the Unified Health System (SUS - *Sistema Único de Saúde*) Information and IT Department (DATASUS), 777,030 people were admitted to hospital because of CVD in Brazil from 2012 to 2022.^6^ National studies show that the majority of patients with varicose veins undergoing surgery are women (61%), while men account for approximately 38% of cases.^7^ In Brazil, this prevalence can be as high as 50% of the population and CVD is the ranked 14th among causes of work absenteeism and 32nd among causes of disability retirement.^7,8^

The main risk factors for development of this condition are remaining in the same position for prolonged periods, prior surgery, LE traumas, inactivity, wearing footwear with heels, obesity, and pregnancy.^1^

The clinical manifestations of CVD can be classified using the Clinical manifestations, Etiologic factors, Anatomic distribution of disease, and Pathophysiologic findings (CEAP) classification. According to the 2020 revision of this classification, clinical signs are categorized into seven classes: Class C0 - no visible or palpable signs of venous disease; Class C1 - telangiectasis (≤1 mm) or reticular veins (1-3 mm); Class C2 - varicose veins (≥3 mm); Class C3 - edema; Class C4 – skin and tissue changes secondary to venous disease (C4a - pigmentation or venous eczema and C4b - lipodermatosclerosis, atrophie blanche, or ochrodermatitis); Class C5 - healed venous ulcer; and Class C6 – active venous ulcer (C6r when recurrent). The updated classification also recommends appending the letter “S” for symptomatic cases or “A” for asymptomatic cases after the clinical class (e.g.: C2S).^3,5,9^

Signs such as varicose veins, edema, trophic skin changes, and ulcers and symptoms such as pain, cramps, itching, feelings of heaviness in the legs, burning, and throbbing are often associated with limitations to daily activities, impaired functional performance, psychological problems, and changes to perceived health status.^3,10,11,12^ Moreover, if CVD is not treated properly, it can progress to complications such as edema, constant pain, hyperpigmentation, venous eczema, thrombophlebitis, ulcer, hemorrhage, and dermatofibrosis.^7,13^

CVD is primarily diagnosed clinically, by means of patient history and physical examination. History includes the patient’s complaints and the duration of symptoms; characteristics of prior diseases (especially venous thrombosis); history of limb traumas; and presence of varicose disease. The main symptoms include feelings of heaviness and pain in the legs, particularly at the end of the day, in conjunction with itching, as reported by some patients.^14^

Physical examination may reveal signs such as hyperpigmentation (when hemoglobin that builds up inside tissues becomes hemosiderin, which colors the skin brown), lipodermatosclerosis (a change caused by progressive substitution of the skin and subcutaneous tissue by fibrosis), pitting edema (more extensive on the symptomatic leg), varicose veins, nevi, increased limb length, and varicose veins in atypical places.^14^ Advances in the vascular laboratory include use of color Doppler ultrasound as a supplementary examination for assessing venous valve incompetence or presence of chronic obstruction.^15,16,17^ This is a rapid, reasonably-priced, noninvasive method with 92% sensitivity and 73% specificity for detection of venous reflux, when compared with descending phlebography.^15,17^

Considering the above, CVD is understood to be a severe public health problem that directly affects occupational productive capacity,^1,18^ significantly reduces the quality of life of people with the disease, and potentially causes psychological changes such as sadness, depression, irritability, worry about appearance, and social isolation.^1,3,19^ Thus, the objective of this study was to analyze temporal trends in hospital admissions for CVD in Brazil from 2014 to 2023.

## METHODS

This ecological study of a temporal series of hospital admissions for CVD in Brazil was conducted using information from the Hospital Information System (SIH- *Sistema de Informação Hospitalar*)^20^ , a public domain database managed by the Information and IT Department (DATASUS) of the Unified Health System (SUS - *Sistema Único de Saúde*), downloaded in the Comma-Separated Values (CSV) format.

The study analyzed hospital admissions for chronic venous disease among the population aged from 20 to 80 years, during the period from 2014 to 2023, in Brazil and its administrative regions. Data were extracted from the database using International Classification of Diseases (ICD-10) nomenclature, seeking the following codes: Phlebitis, thrombophlebitis, emboli and venous thrombosis; Varicose veins of lower extremities; I80.0 – Phlebitis and thrombophlebitis of superficial vessels of lower extremities; I80.1 – Phlebitis and thrombophlebitis of femoral vein; I80.2 – Phlebitis and thrombophlebitis of other and unspecified deep vessels of lower extremities; I80.3 – Phlebitis and thrombophlebitis of lower extremities, unspecified; I80.8 – Phlebitis and thrombophlebitis of other sites; I80.9 – Phlebitis and thrombophlebitis of unspecified site; I83.0 – Varicose veins of lower extremities with ulcer; I83.1 – Varicose veins of lower extremities with inflammation; I83.2 – Varicose veins of lower extremities with both ulcer and inflammation; I83.9 – Asymptomatic varicose veins of lower extremities.

Rates of admission were defined using population data from the 2060 projection published by the Brazilian Institute of Geography and Statistics (IBGE - Instituto Brasileiro de Geografia e Estatística)^21^ and calculated as the ratio of the number of admissions for phlebitis, thrombophlebitis, emboli and venous thrombosis, and varicose veins of the lower extremities to the population. Data were stratified by country (Brazil), region of Brazil, sex (male and female), and age group by sex and are expressed as admissions per 100 thousand inhabitants.

The analysis of temporal trends was conducted using simple linear regression, considering the mean annual variations in rates (β) and their respective 95% confidence intervals (95%CI) and the percentage variation (PV) in rates from the first (2014) to the last (2023) years in the series. Results with p < 0.05 were considered statistically significant. The Statistical Package for the Social Sciences (SPSS), version 18.0 (Chicago: SPSS Inc; 2018), was used to process data and for statistical analysis.

For this method, standardized hospital admission coefficients were considered dependent variables, while the calendar years of the study period were the independent variable. Thus, the model estimated has the Formula 1:


Y = b0 + b1X (1)

where Y = standardized coefficient, b0 = mean coefficient for the period, b1 = mean annual increment, and X = year.

This study complies with the ethical principles set out in National Health Council (*Conselho Nacional de Saúde*) Resolution n° 510/2016. Since this study is based entirely on public domain secondary data, Research Ethics Committee appraisal was unnecessary. The authors have no conflicts of interest to declare.

## RESULTS

**Figure 1:**
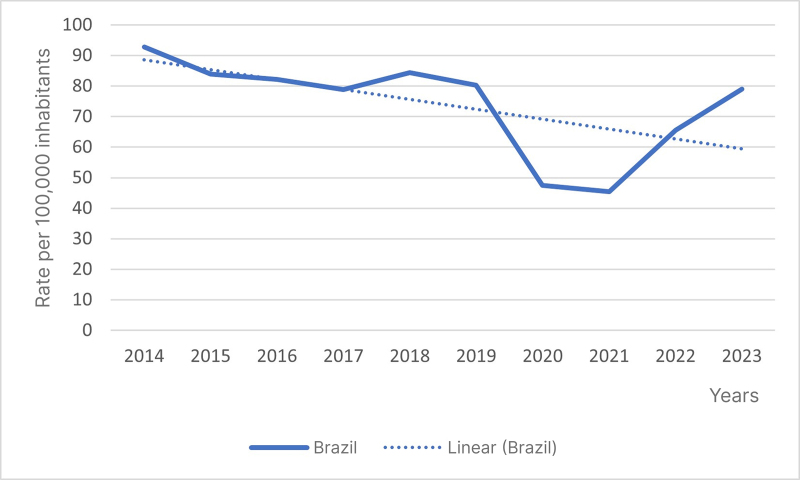
General trend in hospital admissions for chronic venous disease in Brazil from 2014 to 2023 (β = -3.233; p = 0.060). Source: The authors, 2024.

A total of 1,091,733 hospitalizations for CVD in Brazil from 2014 to 2023 were analyzed. The overall rate showed a stable trend (β *=* -3.233*;* p = 0.060) during the study period, with a mean rate of 73.98 admissions per 100 thousand inhabitants (Figure 1).

The analysis per regions of Brazil revealed reductions in the rates of admission for CVD in the Northeast (β = -1.275; *p* = 0.042), Southeast (β = -4.262; *p* = 0.044), and South (β = -7.282; p = 0.012) regions, where the mean rates observed were 37.24, 93.89, and 111.68 admissions per 100 thousand inhabitants, respectively. The percentage reductions from the first (2014) to the last year (2024) were 23.60% in the Northeast, 18.76% in the Southeast, and 31.36% in the South. In contrast, the North and Midwest regions showed stable rates during the period analyzed (Table 1).

**Table 1:** Temporal trends in hospital admission for chronic venous disease by regions of Brazil, age group, and sex, from 2014 to 2023.

**Variables**	**Mean rate** *	**PV (%)** †	**MAV** ‡ **(β)**	**95%CI of MAV** §	**Value de *p*** ||	**Trend**
**Regions of Brazil**						
North	35.46	175.21	2.910	-5.149 to 10.969	0.429	Stability
Northeast	37.24	-23.60	-1.275	-2.493 to -0.058	0.042	Reduction
Southeast	93.89	-18.76	-4.262	-8.371 to -0.154	0.044	Reduction
South	111.68	-31.36	-7.282	-12.439 to -2.125	0.012	Reduction
Midwest	53.14	-8.95	-2.051	-4.609 to 0.507	0.102	Stability
**Age groups Males**						
20 to 29 years	9.82	-25.83	-0.484	-0.785 to -0.182	0.006	Reduction
30 to 39 years	23.36	-25.96	-1.251	-2.068 to -0.434	0.008	Reduction
40 to 49 years	45.18	-21.89	-2.122	-3.810 to -0.434	0.020	Reduction
50 to 59 years	72.62	-13.15	-2.644	-5.250 to -0.038	0.047	Reduction
60 to 69 years	95.59	-14.57	-3.206	-6.104 to -0.309	0.034	Reduction
70 to 79 years	103.03	-23.22	-3.875	-6.251 to -1.499	0.006	Reduction
80 years or over	108.65	-28.57	-4.533	-6.365 to -2.700	<0.001	Reduction
**Age groups Females**						
20 to 29 years	22.88	-42.49	-1.625	-2.307 to -0.943	0.001	Reduction
30 to 39 years	71.16	-41.57	-6.138	-9.524 to -2.753	0.003	Reduction
40 to 49 years	132.20	-22.32	-7.837	-14.800 to -0.874	0.032	Reduction
50 to 59 years	160.02	-7.58	-7.351	-17.299 to 2.597	0.127	Stability
60 to 69 years	158.90	-4.97	-6.250	-15.394 to 2.894	0.154	Stability
70 to 79 years	121.21	-13.35	-4.686	-9.307 to -0.065	0.048	Reduction
80 years or over	119.50	-27.73	-5.218	-7.689 to -2.746	0.001	Reduction

* Mean rate – mean of rates for the period.

† PV –percentage variation in rates from the first (2014) to the last (2023) year.

‡ MAV (β) – Mean Annual Variation (MAV) – Calculated by linear regression.

§ 95%CI of MAV – 95% confidence interval of the Mean Annual Variation.

|| P values < 0.05 considered statistically significant.

Source: The authors, 2024.

**Figure 2:**
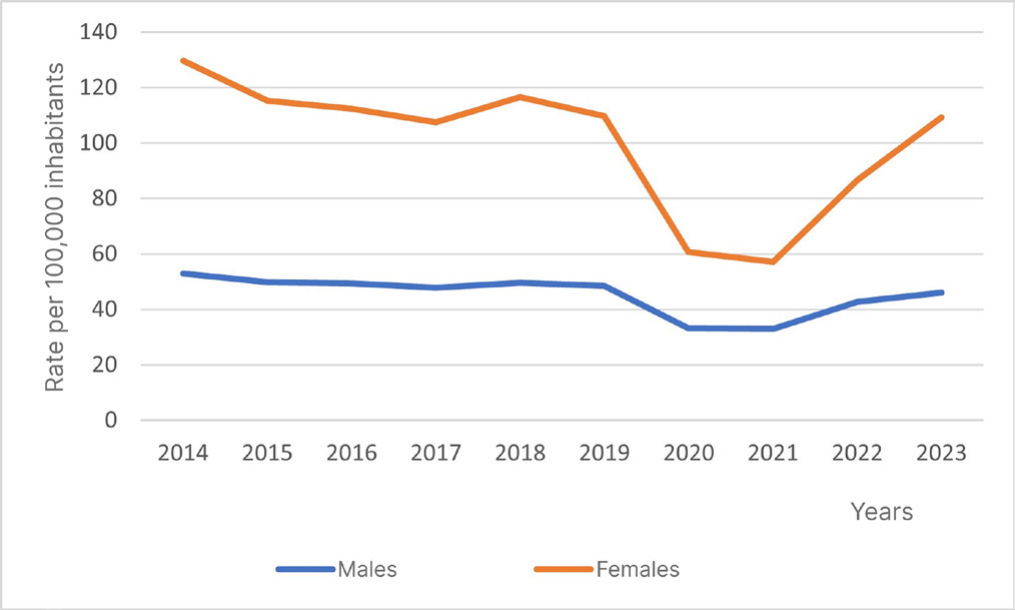
Temporal trend in hospital admissions for chronic venous disease in Brazil from 2014 to 2023, by sex. Source: The authors, 2024.

The analysis of admissions for CVD by sex revealed stable rates over the period analyzed, for both males (β -1.439; p 0.052) and females (β -4.892; p 0.063), with mean rates of 45.29 and 100.50 admissions per 100,000 inhabitants, respectively. Notwithstanding, there was a significant reduction during the period from 2019 to 2021, primarily caused by the COVID-19 pandemic, followed by a significant increase after 2021 (Figure 2).

Among males, reductions were observed in all of the age groups analyzed (20 to 80 years or over). The Mean Annual Variation (MAV) increased progressively as age increased, with mean rates ranging from 9.82 to 108.65 admissions for every 100,000 men. These equate to reductions of 13.15% to 28.57% in the rates from the first to the last years analyzed (Table 1).

Among females, reductions were observed in the age groups 20 to 29 years (β = -1.625; p = 0.001), 30 to 39 years (β = -6.138; p = 0.003), 40 to 49 years (β *=* -7.837; p = 0.032), 70 to 79 years (β *=* -4.686; p = 0.048), and 80 years or over (β *=* -5.218; *p* = 0.001), in which mean rates were 22.88, 71.16, 132.20, 121.21, and 119.50 per 100,000 women respectively, with reductions in the rates of admission for CVD from the first to the last year analyzed of 42.49%, 41.57%, 22.32%, 13.35%, and 27.73%. The rates in the female age groups 50 to 59 years and 60 to 69 years were stable, with no significant research data on prevalence in these age groups (Table 1).

## DISCUSSION

The analysis of rates of admission for chronic venous diseases (CVD) revealed that numbers have stabilized, contributing to averting an overload of the Brazilian health system related to the condition. While studies indicate an alarming tendency to increase in rates of admission because of complications associated with CVD over recent years, this was not confirmed in the current situation in Brazil.

The comparison of statistics extracted from the DATASUS database indicates that admissions for CVD in Brazil have stabilized over time, which contrasts with the literature. However, it is necessary to take account of the vast harm and research deficits left as sequelae of the pandemic in Brazil. According to the literature, the increase in rates of admission would be because of both population aging and also because of the increased prevalence of risk factors and possible changes in standards for diagnosis and treatment.^1-13^ The literature emphasizes that CVD does not only impose a substantial financial burden on the health system, but also has negative impacts on the quality of life of patients, increasing their risk of morbidity and mortality.^1,22,23,24^ Comprehension of these patterns of admission is crucial to guide public policies targeting effective prevention and management of CVD, aiming to mitigate the impact on both the individual and collective levels.

Estimates of the prevalence of varicose veins and CVD vary significantly, with studies showing higher prevalence of varicose veins among women than men. This can be attributed to several biological, hormonal, and behavioral factors. Studies indicate that the prevalence of varicose veins can range from less than 1% to 73% in women, compared to a range of 2% to 56% in men.^25^ The greater prevalence among women naturally creates opportunities for the higher number of admissions.

One factor that plays a significant role in development of varicose veins and CVD in women are female hormones such as estrogen and progesterone. These hormones can weaken the venous walls and valves, increasing women’s susceptibility to CVD.^25^ Another factor observed to contribute to the prevalence among women is their greater propensity to seek medical treatment for symptoms of CVD because of greater sensitivity to pain or discomfort compared with men.^25^

During pregnancy, pathophysiologic changes occur in the hemostatic system, provoking a hypercoagulable state that significantly raises the risk of venous thromboembolic events (VTE) among expectant mothers. Studies demonstrate incidence of 0.6 to 1.7 cases of VTE per 1,000 pregnancies.^26^ While pregnancy is an established risk factor, the higher prevalence of CVDs among women remains even among those who are not pregnant. Studies demonstrate that use of hormonal contraceptives, which is particularly relevant in Brazil, where use is widespread, are associated with an increase of up to three times the risk of development of CVD.^27^ In one article that presented a critical analysis of epidemiological studies, it was shown that menopausal hormone replacement therapy is also associated with a significant increase in the risk of venous thromboembolism in women, increasing relative risk by 2.1 to 3.5 times, depending on age group and duration of treatment.^28^ These findings confirm data from the SUS SIH, which show that women account for >50% of admissions for deep venous thrombosis and phlebitis, even after exclusion of cases related to pregnancy.^6^ This epidemiological profile highlights the multifactorial nature of CVD etiology, which involves exogenous hormonal factors, genetic predisposition, and behavioral components, such as inactivity and obesity.^25^

In the data analyzed for this study, the reduction of rates of admission during 2020, 2021, and 2022 is clear, with cases increasing once more in 2023. During this specific period, the World Health Organization (WHO) declared a Public Health Emergency of International Concern, better known by most as the COVID-19 pandemic, starting in March 2020 and ending in May 2023.^29^ In this scenario, it is understood that many people avoided seeking medical attention because of fear of contracting COVID-19 in hospital settings, which could have contributed to under-notification and a reduction in admissions.^30^ Moreover, just as the population in general withdrew and avoided hospitals because of fear, the hospitals and clinics themselves redirected resources to treatment of patients with COVID-19, suspending or delaying elective treatments and non-emergency procedures, including those for CVD.^31^

While this national analysis of Brazil did not demonstrate a statistically significant reduction in admissions for CVD (β = -3.233; p = 0.060), there was a mean annual reduction of 3.23 hospitalizations per 100 thousand inhabitants, indicating a relevant practical trend, close to the threshold of significance. This pattern is underscored by significant reductions in three of the five regions analyzed (South, Southeast, and Northeast), while the North and Midwest regions and specific subsets of patients (such as women aged 50 - 69 years) remained stable, reflecting possible inequalities in the effectiveness of health care policies. However, the wider context still shows evidence of the considerable impact of CVD on the Brazilian system, with more than one million admissions registered, in addition to the additional challenges imposed by crises such as COVID-19, which exposed the system’s vulnerability to external factors. These findings are aligned with recent studies that highlight the growing complexity of clinical management and the costs associated with vascular diseases (venous and arterial), even in the context of variations in hospital admission rates.^32,33^ This scenario underscores the urgent need for strategies targeting prevention, early diagnosis, and adequate treatment, prioritizing populations and regions without significant reductions, in addition to the need for investigation of the underlying social and clinical determinants of the inequalities observed. Such measures are essential, not only to reduce demand for hospital and emergency services, but also to improve patients’ quality of life.

In the realm of genetics, epidemiological studies in Brazil, such as one conducted in the state of Minas Gerais, emphasize the importance of genetic tests for identification of individuals at risk and for effective clinical management of thrombosis and CVD.^34^ Presence of genetic mutations, such as factor V Leiden and the G20210A mutation of the prothrombin gene, combined with hormonal and physiological factors associated with pregnancy, also contribute to increased risk of venous thrombosis among women.^34^

The findings of this study do not merely reflect the epidemiological burden of chronic venous diseases, they are also evidence of the social and economic impact of these conditions on the Brazilian health system. Understanding these trends is crucial to guide public policies targeting vascular health with the objective of improving the quality of life of patients and the efficiency of hospital resource allocation.

It is also essential to consider the impact of the COVID-19 pandemic on rates of admission, underscoring the importance of continuous surveillance and adaptive strategies to face future challenges related to CVD. Such strategies should prioritize screening and reduction of risk factors, aiming at prevention of the development and complications of chronic vascular comorbidity.

This study has contributed to understanding of the epidemiology of CVD in Brazil and highlights the need for continuous investments in vascular health, focused on prevention, early diagnosis, and correct treatment, aiming to reduce the burden of these conditions of public health and improve the wellbeing of affected patients.

## CONCLUSIONS

The study results revealed stability in the overall rate of hospital admissions for CVD in Brazil from 2014 to 2023, although with significant regional and age-related variations, with a significant reduction during the COVID-19 pandemic and a significant increase after the end of the pandemic in 2023.

Although reductions were observed in the rates of admission in the Northeast, Southeast, and South regions, the falls were borderline in terms of the statistical analysis. This, therefore, does not eliminate the need for improved strategies for prevention and treatment of CVD in these regions or for possible changes in the standards of health and the health services provided. In turn, rates in the North and Midwest remained stable, showing an even greater need for interventions to achieve reductions in admission rates.

The analysis by sex and age group highlighted important reductions in hospital admission rates among both sexes, especially in the younger female population. However, the stability observed among women in the age groups from 50 to 69 years indicates a need for attention and for strategies targeting this population.

## Data Availability

Disponíveis em repositório público com DOI: “Os dados que fundamentam os achados deste estudo estão disponíveis no repositório público do Departamento de Informática do Sistema Único de Saúde – DATASUS (http://datasus.saude.gov.br)”.
