# Supplementary material for: Temporal trend of hospitalization for chronic venous disease in Brazil from 2014 to 2023
Source: J Vasc Bras. 2025 Oct 27;24:e20240136. doi: 10.1590/1677-5449.202401362 (PMC12642923; doi:10.1590/1677-5449.202401362)
Supplement: Translation

# Translation: Tendência temporal de hospitalização por doença venosa crônica no Brasil entre 2014 e 2023

**DOI:** 10.1590/1677-5449.202401361

**Authors:** Lucas Sgrott Simão Flausino, Douglas Fernando Kunkel, João Guilherme Cardoso, Lucas Napoli Cordeiro, Kailan Goulart da Silveira, Gabriele Sousa Silval, Maysa Melo Henkes, Fabiana Oenning da Gama

**Affiliations:** 1 Universidade do Sul de Santa Catarina – UNISUL, Palhoça, SC, Brasil.

**Keywords:** doenças vasculares, varizes, tromboflebite, trombose, hospitalização, epidemiologia

## Abstract

**Contexto:**

A doença venosa crônica dos membros inferiores (DVC) é uma enfermidade de importante prevalência e taxa de hospitalização.

**Objetivos:**

Analisar a tendência temporal de hospitalizações por DVC no Brasil entre 2014 e 2023.

**Métodos:**

Estudo ecológico de séries temporais de hospitalização por DVC utilizando dados do Sistema de Informação Hospitalar do Sistema Único de Saúde. Utilizou-se regressão linear simples, com variação média anual das taxas (β) e variação percentual, considerando p < 0,05.

**Resultados:**

Analisaram-se 1.091.733 internações por DVC no Brasil no período estudado. A taxa geral (β = -3,233; p = 0,060) apresentou tendência de estabilidade, com média de 73,98 internações por 100 mil habitantes. Houve redução estatisticamente significativa no Nordeste (β = -1,275; p = 0,042), Sudeste (β = -4,262; p = 0,044) e Sul (β = -7,282; p = 0,012). Observou-se estabilidade tanto em homens (β = -1,439; p = 0,052) quanto em mulheres (β = -4,892; p = 0,063), com taxas mais elevadas entre estas. Homens de 20 a 80 anos ou mais (p < 0,05) e mulheres de 20 a 49 e 70 anos ou mais (p < 0,05) apresentaram redução significativa.

**Conclusões:**

A taxa geral de internações por DVC no Brasil mostrou-se estável. Houve redução nas regiões Nordeste, Sudeste e Sul, e estabilidade nas demais. Ambos os sexos apresentaram tendência estável. Verificou-se redução entre homens de 20 a 80 anos ou mais e mulheres de 20 a 49 e 70 anos ou mais, com estabilidade apenas em mulheres de 50 a 69 anos.

## INTRODUÇÃO

A doença venosa crônica dos membros inferiores (DVC) destaca-se atualmente como uma das enfermidades crônicas de maior prevalência e incidência, além de elevada morbidade, na população^1,2^ .

A DVC é definida como uma disfunção no sistema venoso causada por incompetência valvular associada ou não a obstrução do fluxo venoso. Essa anormalidade venosa pode ser congênita ou adquirida, podendo acometer tanto o sistema venoso superficial quanto o profundo^3-5^ .

Segundo o Departamento de Informação e Informática do Sistema Único de Saúde (DATASUS) do Brasil, entre 2012 e 2022 foram internadas 777.030 pessoas por DVC no Brasil^6^ . Estudos nacionais mostram que a maioria dos portadores de varizes submetidos a cirurgia são mulheres (61%), enquanto os homens representam cerca de 38% dos casos^7^ . No Brasil, essa prevalência pode chegar a 50% da população, sendo a 14ª causa de absenteísmo no trabalho e a 32ª causa de aposentadoria por invalidez^7,8^ .

Os principais fatores de risco para o desenvolvimento dessa condição são permanência na mesma postura por períodos prolongados, cirurgias prévias, traumatismo dos membros inferiores (MMII), sedentarismo, uso de calçados com saltos, obesidade e gestação^1^ .

As manifestações clínicas decorrentes da DVC podem ser classificadas com base na classificação *Clinical manifestations, Etiologic factors, Anatomic distribution of disease, Pathophysiologic findings* (CEAP). De acordo com a revisão de 2020 dessa classificação, os sinais clínicos são categorizados em sete classes: Classe C0 - ausência de sinais visíveis ou palpáveis de doença venosa; Classe C1 - telangiectasias (≤1 mm) ou veias reticulares (1-3 mm); Classe C2 - veias varicosas (≥3 mm); Classe C3 - edema; Classe C4 - alterações cutâneas e teciduais decorrentes da doença venosa (C4a - pigmentação ou eczema venoso e C4b - lipodermatoesclerose, atrofia branca ou dermatite ocre); Classe C5 - úlcera venosa cicatrizada; e Classe C6 - úlcera venosa ativa (sendo C6r quando recorrente). A classificação atualizada também recomenda a adição da letra “S” para casos sintomáticos ou “A” para assintomáticos após a classe clínica (ex.: C2S)^3,5,9^ .

Sinais como veias varicosas, edema, alterações tróficas da pele e úlceras, assim como sintomas como dor, câimbras, prurido, sensação de peso nas pernas, queimação e latejamento, estão frequentemente associados a limitações nas atividades diárias, prejuízo no desempenho funcional, alterações psicológicas e mudança na percepção do estado de saúde^3,10-12^ . Ademais, a DVC, se não adequadamente tratada, pode progredir e levar a complicações como edema, dor constante, hiperpigmentação, eczema venoso, tromboflebite, úlcera, hemorragia e dermatofibrose^7,13^ .

O diagnóstico da DVC é, principalmente, clínico, realizado por meio da anamnese e exame físico. Na anamnese consideram-se a queixa do paciente e a duração dos sintomas; a caracterização de doenças anteriores (especialmente trombose venosa); traumatismos prévios dos membros; e a existência de doença varicosa. Entre os sintomas, destaca-se a sensação de peso e dor nas pernas, sobretudo ao final do dia, acompanhada de prurido, conforme relatado por alguns pacientes^14^ .

Ao exame físico, podem ser observados sinais como hiperpigmentação (quando a hemoglobina que permanece no interior tissular se transforma em hemossiderina, que dá coloração castanha à pele), lipodermatoesclerose (alteração devido à substituição progressiva da pele e do tecido subcutâneo pela fibrose), edema depressível (maior na perna sintomática), veias varicosas, nevos, aumento do comprimento do membro e varizes de localização atípica^14^ . Com os avanços nas tecnologias do laboratório vascular, emprega-se também o eco-color Doppler como exame complementar para avaliar a incompetência valvar venosa ou a presença de obstrução crônica^15-17^ . Trata-se de um método rápido, não invasivo, de custo razoável, com sensibilidade de 92% e especificidade de 73% para detecção de refluxo venoso, quando comparado à flebografia descendente^15,17^ .

Diante disso, entende-se a DVC como um grave problema de saúde pública que afeta diretamente a capacidade produtiva de trabalho^1,18^ , reduz de modo significativo a qualidade de vida dos portadores da doença e leva a potenciais alterações psicológicas como tristeza, depressão, irritabilidade, preocupação com a aparência e isolamento social^1,3,19^ . Assim, o presente estudo teve como objetivo analisar a tendência temporal de hospitalização por DVC no Brasil entre 2014 e 2023.

## MÉTODOS

Este estudo ecológico de séries temporais de hospitalização por DVC no Brasil foi realizado com base no banco de dados de domínio público Sistema de Informação Hospitalar (SIH)^20^ , disponibilizado pelo Departamento de Informação e Informática do Sistema Único de Saúde (DATASUS), com cópia no formato *Comma-Separated Value* (CSV).

Foram analisadas as internações hospitalares por doença venosa crônica na população com idade entre 20 e 80 anos, no período de 2014 a 2023, no Brasil e em suas regiões. Os dados foram extraídos do banco de dados conforme a nomenclatura da Classificação Internacional de Doenças (CID-10) considerando os seguintes códigos: Flebite, tromboflebite, embolia e trombose venosa; Veias varicosas das extremidades inferiores; I80.0 – Flebite e tromboflebite dos vasos superficiais dos membros inferiores; I80.1 – Flebite e tromboflebite da veia femoral; I80.2 – Flebite e tromboflebite de outros vasos profundos dos membros inferiores; I80.3 – Flebite e tromboflebite dos membros inferiores, não especificada; I80.8 – Flebite e tromboflebite de outras localizações; I80.9 – Flebite e tromboflebite de localização não especificada; I83.0 – Varizes dos membros inferiores com úlcera; I83.1 – Varizes dos membros inferiores com inflamação; I83.2 – Varizes dos membros inferiores com úlcera e inflamação; I83.9 – Varizes dos membros inferiores sem úlcera ou inflamação.

As taxas de internação foram definidas com base nos dados populacionais da projeção de 2060 do Instituto Brasileiro de Geografia e Estatística (IBGE)^21^ e calculadas por meio da razão entre o número de internações por flebite, tromboflebite, embolia e trombose venosa e veias varicosas das extremidades inferiores e a população. Os dados foram estratificados por país (Brasil), regiões brasileiras, sexo (masculino e feminino) e faixas etárias por sexo, sendo expressos por 100 mil habitantes.

A análise de tendência temporal foi realizada por meio de regressão linear simples, considerando a variação média anual das taxas (β) com seus respectivos intervalos de confiança de 95% (IC95%) e a variação percentual (VP) entre as taxas do primeiro (2014) e último ano (2023). Valores de p < 0,05 foram considerados estatisticamente significativos. Para processamento dos dados e análise estatística, utilizou-se o programa *Statistical Package for the Social Sciences* (SPSS), versão 18.0 (Chicago: SPSS Inc; 2018).

Nesse método, os coeficientes de internação padronizados foram considerados variáveis dependentes, enquanto os anos-calendário do estudo constituíram a variável independente. Assim, o modelo estimado seguiu a Fórmula 1:


Y = b0 + b1X (1)

onde Y = coeficiente padronizado, b0 = coeficiente médio do período, b1 = incremento anual médio e X = ano.

Este estudo obedeceu aos preceitos éticos da Resolução n° 510/2016 do Conselho Nacional de Saúde. Por tratar-se de pesquisa baseada em dados secundários de domínio público, não foi necessária a avaliação do Comitê de Ética em Pesquisa. Os autores declaram ausência de conflitos de interesse.

## RESULTADOS

Foram analisadas 1.091.733 hospitalizações por DVC no Brasil entre os anos de 2014 e 2023. Verificou-se tendência de estabilidade na taxa geral (β *=* -3,233*;* p = 0,060) no período estudado, com taxa média de 73,98 internações por 100 mil habitantes (Figura 1).

**Figura 1:** Tendência geral de internações por doença venosa crônica no Brasil de 2014 a 2023 (β = -3,233; p = 0,060). Fonte: Elaboração dos autores, 2024.

Na análise por regiões do Brasil, observou-se redução das taxas de internação por DVC nas regiões Nordeste (β = -1,275; *p* = 0,042), Sudeste (β = -4,262; *p* = 0,044) e Sul (β = -7,282; p = 0,012). As taxas médias observadas foram de 37,24, 93,89 e 111,68 internações por 100 mil habitantes, respectivamente. As reduções percentuais entre o primeiro (2014) e último ano (2024) foram de 23,60% no Nordeste, 18,76% no Sudeste e 31,36% no Sul. Por outro lado, as regiões Norte e Centro-Oeste apresentaram estabilidade no período analisado (Tabela 1).

**Tabela 1:** Tendência temporal de internação por doença venosa crônica conforme região do Brasil, faixa etária e sexo, de 2014 a 2023.

**Variáveis**	**Taxa Média** *	**VP (%)** †	**VMA** ‡ **(β)**	**IC95% da VMA** §	**Valor de *p*** ||	**Tendência**
**Regiões do Brasil**						
Norte	35,46	175,21	2,910	-5,149 a 10,969	0,429	Estabilidade
Nordeste	37,24	-23,60	-1,275	-2,493 a -0,058	0,042	Redução
Sudeste	93,89	-18,76	-4,262	-8,371 a -0,154	0,044	Redução
Sul	111,68	-31,36	-7,282	-12,439 a -2,125	0,012	Redução
Centro-Oeste	53,14	-8,95	-2,051	-4,609 a 0,507	0,102	Estabilidade
**Faixas etárias Sexo masculino**						
20 a 29 anos	9,82	-25,83	-0,484	-0,785 a -0,182	0,006	Redução
30 a 39 anos	23,36	-25,96	-1,251	-2,068 a -0,434	0,008	Redução
40 a 49 anos	45,18	-21,89	-2,122	-3,810 a -0,434	0,020	Redução
50 a 59 anos	72,62	-13,15	-2,644	-5,250 a -0,038	0,047	Redução
60 a 69 anos	95,59	-14,57	-3,206	-6,104 A -0,309	0,034	Redução
70 a 79 anos	103,03	-23,22	-3,875	-6,251 a -1,499	0,006	Redução
80 anos mais	108,65	-28,57	-4,533	-6,365 a -2,700	<0,001	Redução
**Faixas etárias Sexo feminino**						
20 a 29 anos	22,88	-42,49	-1,625	-2,307 a -0,943	0,001	Redução
30 a 39 anos	71,16	-41,57	-6,138	-9,524 a -2,753	0,003	Redução
40 a 49 anos	132,20	-22,32	-7,837	-14,800 a -0,874	0,032	Redução
50 a 59 anos	160,02	-7,58	-7,351	-17,299 a 2,597	0,127	Estabilidade
60 a 69 anos	158,90	-4,97	-6,250	-15,394 a 2,894	0,154	Estabilidade
70 a 79 anos	121,21	-13,35	-4,686	-9,307 a -0,065	0,048	Redução
80 anos mais	119,50	-27,73	-5,218	-7,689 a -2,746	0,001	Redução

* Taxa Média – média das taxas do período.

† VP – variação percentual entre as taxas do primeiro (2014) e último ano (2023).

‡ VMA (β) – Variação Média Anual (VMA) - Calculada por Regressão Linear.

§ IC95% da VMA – Intervalo de Confiança de 95% da Variação Média Anual.

|| valor de p < 0,05 considerado estatisticamente significante.

Fonte: Elaboração dos autores, 2024.

Na análise por sexo das internações por DVC, verificou-se estabilidade nas taxas ao longo do período avaliado, tanto para o sexo masculino (β -1,439; p 0,052) quanto para o feminino (β -4,892; p 0,063), com taxa média de 45,29 e 100,50 internações por 100.000 habitantes, respectivamente. Contudo, identificou-se um decréscimo significativo no período de 2019 a 2021, causado principalmente pela pandemia de COVID-19, seguido por um aumento significativo após o ano de 2021 (Figura 2).

**Figura 2:** Tendência temporal de internações por doença venosa crônica de 2014 a 2023, dividida por sexos. Fonte: Elaboração dos autores, 2024.

No sexo masculino, foi observada redução em todas as faixas etárias analisadas (20 a 80 anos ou mais). A Variação Média Anual (VMA) apresentou incremento progressivo conforme o aumento da faixa etária, com taxas médias que variaram de 9,82 a 108,65 internações a cada 100.000 homens. Isso significou uma redução de 13,15% a 28,57% nas taxas entre o primeiro e último ano analisados (Tabela 1).

No sexo feminino, foi observada redução nas faixas etárias de 20 a 29 anos (β = -1,625; p = 0,001), 30 a 39 anos (β = -6,138; p = 0,003), 40 a 49 anos (β *=* -7,837; p = 0,032), 70 a 79 anos (β *=* -4,686; p = 0,048) e naquelas acima de 80 anos (β *=* -5,218; *p* = 0,001). Nessas faixas, foram verificadas taxas médias de 22,88, 71,16, 132,20, 121,21 e 119,50 por 100.000 mulheres, respectivamente, bem como redução das taxas de internação por DVC de 42,49%, 41,57%, 22,32%, 13,35% e 27,73% entre o primeiro e último ano analisados. As taxas nas faixas etárias femininas de 50 a 59 anos e 60 a 69 anos apresentaram estabilidade, não havendo dados significativos de pesquisa sobre a prevalência nessas faixas (Tabela 1).

## DISCUSSÃO

A análise das taxas de internação por doenças venosas crônicas (DVC) revela uma estabilização nos números, o que contribui para evitar a sobrecarga no sistema de saúde brasileiro relacionada a essa condição. Embora estudos apontem uma tendência alarmante de aumento nas taxas de internação devido a complicações associadas às DVC ao longo dos últimos anos, isso não é verificado na realidade brasileira atual.

A comparação das estatísticas levantadas com base nos dados do DATASUS aponta para uma estabilização nas internações por DVC no Brasil ao longo do tempo, o que vai de encontro à literatura. No entanto, é preciso levar em conta os vastos prejuízos e déficits de pesquisas deixados como sequela da pandemia no país. Segundo a literatura, o aumento nas taxas de internação seria explicado não apenas pelo envelhecimento populacional, mas também pelo aumento da prevalência de fatores de risco e possíveis mudanças nos padrões de diagnóstico e tratamento^1-13^ . A literatura destaca que as DVC não apenas geram um ônus econômico substancial para o sistema de saúde, mas também impactam negativamente na qualidade de vida dos pacientes, aumentando o risco de morbidade e mortalidade^1,22-24^ . A compreensão desses padrões de internação é crucial para orientar políticas públicas voltadas à prevenção e manejo eficaz das DVC, visando mitigar seu impacto tanto a nível individual quanto coletivo.

As estimativas da prevalência de varizes e DVC variam significativamente, com estudos mostrando uma maior prevalência de varizes em mulheres do que em homens. Isso pode ser atribuído a diversos fatores biológicos, hormonais e comportamentais. Estudos indicam que a prevalência de varizes pode variar de menos de 1% a 73% em mulheres, comparado a uma variação de 2% a 56% em homens^25^ . A maior prevalência em mulheres naturalmente oportuniza um maior número de internações nesta população.

Um dos fatores que desempenham um papel significativo no desenvolvimento de varizes e DVC em mulheres são os hormônios femininos, como o estrogênio e a progesterona. Esses hormônios podem enfraquecer as paredes venosas e as válvulas, aumentando a susceptibilidade das mulheres à DVC^25^ . Outro fator observado que contribui para a prevalência em mulheres foi a maior procura por tratamento médico para sintomas de DVC devido a uma maior sensibilidade à dor ou desconforto em comparação aos homens^25^ .

Durante a gestação, ocorrem alterações fisiopatológicas no sistema hemostático, promovendo um estado de hipercoagulabilidade que eleva significativamente o risco de eventos tromboembólicos venosos (VTE) nas gestantes. Estudos demonstram incidência de 0,6 a 1,7 casos de TEV por 1.000 gestações^26^ . Embora a gravidez seja um fator de risco estabelecido, a maior prevalência de DVCs em mulheres persiste mesmo entre as não gestantes. Estudos demonstram que o uso de anticoncepcionais hormonais, particularmente relevante no contexto brasileiro, onde são amplamente utilizados, está associado a um aumento de até três vezes do risco de desenvolvimento de DVC^27^ . Em um artigo que realizou a análise crítica de estudos epidemiológicos, evidenciou-se que a terapia de reposição hormonal da menopausa (TRHM) também está associada a um aumento significativo do risco de tromboembolismo venoso nas mulheres, elevando o risco relativo em 2,1 a 3,5 vezes conforme a faixa etária e o tempo de tratamento^28^ . Esses achados corroboram os dados do Sistema de Informações Hospitalares do SUS, nos quais mulheres representam >50% das internações por trombose venosa profunda e flebite, mesmo após exclusão de casos gestacionais^6^ . Esse perfil epidemiológico ressalta a natureza multifatorial da etiologia da DVC, que envolve fatores hormonais exógenos, predisposição genética e componentes comportamentais, como sedentarismo e obesidade^25^ .

Nos dados analisados neste estudo, é notória a redução das taxas de internação durante os anos de 2020, 2021 e 2022, com novo aumento dos casos em 2023. Durante esse período em específico, a Organização Mundial da Saúde (OMS) declarou estado de Emergência de Saúde Pública de Importância Internacional (ESPII), conhecido por muitos como a pandemia de COVID-19, com início em março de 2020 e término em maio de 2023^29^ . Diante desse cenário, compreende-se que muitas pessoas evitaram procurar atendimento médico por receio de contrair COVID-19 em ambientes hospitalares, o que pode ter contribuído para a subnotificação e redução nas internações^30^ . Além disso, assim como a população em geral recluiu-se e evitou ambientes hospitalares por medo, os hospitais e clínicas redirecionaram recursos ao tratamento de pacientes com COVID-19, resultando na suspensão ou adiamento de tratamentos eletivos e procedimentos não emergenciais, incluindo os para DVC^31^ .

Embora a análise nacional não tenha demonstrado uma redução estatisticamente significativa nas internações por DVC (β = -3,233; p = 0,060), observou-se um declínio médio anual de 3,23 hospitalizações por 100 mil habitantes, indicando uma tendência prática relevante, próxima ao limiar de significância. Esse padrão é reforçado por quedas significativas em três das cinco regiões analisadas (Sul, Sudeste e Nordeste), enquanto as regiões Norte e Centro-Oeste, assim como subgrupos específicos (como mulheres de 50–69 anos), mantiveram estabilidade, refletindo possíveis desigualdades na efetividade das políticas de saúde. O contexto mais amplo, no entanto, ainda evidencia o impacto expressivo das DVC no sistema brasileiro, com mais de um milhão de internações registradas, além dos desafios adicionais impostos por crises como a COVID-19, que expuseram a vulnerabilidade do sistema a fatores externos. Esses achados estão alinhados com estudos recentes que destacam a crescente complexidade do manejo clínico e dos custos associados às doenças vasculares (venosas e arteriais), mesmo diante de variações nas taxas de hospitalização^32,33^ . Diante desse cenário, reforça-se a urgência de estratégias direcionadas à prevenção, diagnóstico precoce e tratamento adequado, priorizando populações e regiões sem redução significativa, além da investigação dos determinantes sociais e clínicos subjacentes às disparidades observadas. Tais medidas são essenciais não apenas para reduzir a demanda por serviços hospitalares e emergenciais, mas também para melhorar a qualidade de vida dos pacientes.

No âmbito genético, estudos epidemiológicos no Brasil, como o realizado em Minas Gerais, destacam a importância dos testes genéticos na identificação de indivíduos em risco e no manejo clínico eficaz da trombose e da DVC^34^ . A presença de mutações genéticas, como o fator V Leiden e a mutação G20210A no gene da protrombina, combinada a fatores hormonais e fisiológicos associados à gravidez, também contribui para um risco aumentado de trombose venosa em mulheres^34^ .

Os achados deste estudo não apenas refletem a carga epidemiológica das doenças venosas crônicas, mas também evidenciam o impacto econômico e social dessas condições sobre o sistema de saúde brasileiro. A compreensão desses padrões é crucial para orientar políticas públicas voltadas à saúde vascular cujo objetivo seja melhorar a qualidade de vida dos pacientes e a eficiência na alocação de recursos hospitalares.

Também é fundamental considerar o impacto da pandemia de COVID-19 nas taxas de internação, o que sublinha a importância de uma vigilância contínua e de estratégias adaptativas para enfrentar futuros desafíos relacionados à DVC. Tais estratégias devem priorizar o rastreamento e a redução dos fatores de risco, visando à prevenção do desenvolvimento e complicações da comorbidade vascular crônica.

Este estudo contribui para o entendimento da epidemiologia da DVC no Brasil e reforça a necessidade de investimentos contínuos em saúde vascular com foco na prevenção, diagnóstico precoce e tratamento adequado, a fim de reduzir o ônus dessas condições sobre a saúde pública e melhorar o bem-estar dos pacientes afetados.

## CONCLUSÃO

Os resultados revelaram estabilidade na taxa geral de hospitalização por DVC no Brasil entre os anos de 2014 e 2023, embora com variações regionais e etárias significativas, com um decréscimo significativo durante a pandemia de COVID-19 e com um aumento significativo após o fim da pandemia em 2023.

Apesar da redução observada nas taxas de internação nas regiões Nordeste, Sudeste e Sul, as quedas foram limítrofes para análise estatística. Isso, porém, não suspende a necessidade de melhorias nas estratégias de prevenção e tratamento da DVC nesses locais, bem como possíveis mudanças nos padrões de saúde e nos serviços de saúde oferecidos. Já as regiões Norte e Centro-Oeste mantiveram-se estáveis, evidenciando uma necessidade ainda maior de intervenções para que haja redução das internações.

A análise por sexo e faixa etária destacou uma redução importante nas taxas de hospitalização em ambos os sexos, especialmente entre a população jovem do sexo feminino. Contudo, a estabilidade observada em mulheres na faixa etária dos 50 aos 69 anos indica a necessidade de atenção e de estratégias direcionadas a essa população.

## Data Availability

Available in public repository with DOI: “The data supporting the findings of this study are available in the public repository of the Department of Informatics of the Brazilian Unified Health System – DATASUS (http://datasus.saude.gov.br).”
